# Influence of Air-Abrasion Pretreatments and Adhesive Composition on Bond Durability to Natural Sclerotic Dentin

**DOI:** 10.3390/dj14080469

**Published:** 2026-08-02

**Authors:** Silvia del Cid Rodríguez, Carmen Carda Batalla, Rubén Agustín-Panadero, Eva González-Angulo, Juan Luis Román-Rodríguez

**Affiliations:** 1 Private Practice, 29001 Málaga, Spain; 2 Department of Dental Pathology and Therapeutics, Faculty of Medicine and Dentistry, Universitat de València, 46010 Valencia, Spain; 3 Department of Stomatology, Faculty of Medicine and Dentistry, Universitat de València, 46010 Valencia, Spain; ruben.agustin@uv.es (R.A.-P.);

**Keywords:** sclerotic dentin, microtensile bond strength, air-abrasion, aluminum oxide, bioglass, MDP, self-etch adhesive, cervical lesions, adhesion durability

## Abstract

**Background:** Bonding to sclerotic dentin remains one of the most challenging procedures in restorative dentistry due to tubular occlusion and hypermineralization, which limit resin infiltration. This study evaluated the influence of adhesive system, surface treatment, storage time, and substrate position on microtensile bond strength (µTBS) to natural sclerotic dentin. **Methods:** Sixteen extracted human molars with sclerotic dentin were selected and randomly assigned to nine experimental groups according to the adhesive system and surface treatment. Three adhesives were tested: a two-step self-etch adhesive (Clearfil SE Bond 2, Kuraray Noritake Dental, Tokyo, Japan) and two universal adhesives, one containing 10-MDP (G-Premio Bond, GC Corp., Tokyo, Japan) and one without 10-MDP (iBond Universal, Kulzer GmbH, Hanau, Germany). Each adhesive was applied under three conditions: no pretreatment (control), Al_2_O_3_ air-abrasion, and Bioglass 45S5 (ProSylc^®^, Velopex International, London, UK) air-abrasion. Composite build-ups were performed, and the specimens were sectioned into 1 mm^2^ beams for microtensile testing either after 24 h or after 6 months of storage in distilled water at 37 °C of hypertonic solution. The effect of beam position (central vs. peripheral) was also analyzed. Bond strength was measured by µTBS testing, and the results were analyzed using multifactorial ANOVA and Tukey’s test (α = 0.05). Failure modes were examined under stereomicroscopy, whereas optical microscopy was used for qualitative evaluation of representative fractured interfaces. **Results:** Significant main effects were found for adhesive system and surface treatment (*p* < 0.001), with a notable interaction between them (*p* < 0.05). Al_2_O_3_ air-abrasion produced the highest bond strengths, particularly for the self-etch adhesive containing functional monomers (53.4 ± 6.1 MPa), representing a 53% increase over the untreated control. Bioglass air-abrasion did not enhance adhesion and led to irregular hybrid layers. The position of the beams affected only one universal adhesive, with higher µTBS in peripheral regions. After six months of water storage, bond strengths remained stable across most groups, indicating good hydrolytic resistance of the interfaces. **Conclusions:** Both the adhesive system and the surface treatment significantly influenced bonding effectiveness to sclerotic dentin. The combination of Al_2_O_3_ air-abrasion with an MDP-containing self-etch adhesive achieved the most durable and homogeneous bond interface. providing a clinically reliable protocol for restorative treatments in sclerotic dentin. Within the limitations of this in vitro study, this combination may represent a potentially useful strategy for improving adhesion to sclerotic dentin, although further investigations are required to confirm its clinical applicability.

## 1. Introduction

Adhesive dentistry has evolved substantially in recent decades, yet bonding to dentin remains a technically sensitive and multifactorial procedure. Among the various types of dentin, sclerotic dentin is recognized as one of the most difficult substrates for reliable adhesion because of its highly mineralized composition and reduced organic matrix [1,2,3,4,5]. This substrate frequently develops in cervical root areas as a result of physiological aging, abrasion, or erosion, and is characterized by the deposition of intratubular mineral plugs that partially or completely occlude the dentinal tubules [2,3,4]. Such microstructural changes produce a glassy, impermeable surface that limits adhesive penetration, resulting in weak or discontinuous hybrid layers and decreased bond strength [4,5]. The compact and hypermineralized nature of sclerotic dentin hinders the infiltration of resin monomers into the collagen network, which leads to a hybrid layer that is thinner and less homogeneous than in normal dentin [2,4,5]. Consequently, bonds to sclerotic dentin tend to show greater interfacial nanoleakage and reduced mechanical retention. These limitations have motivated the search for strategies that enhance both the chemical and micromechanical aspects of adhesion. The introduction of functional monomers such as 10-methacryloyloxydecyl dihydrogen phosphate (10-MDP) represented a milestone in improving dentin bonding. The MDP molecule is capable of forming stable calcium–phosphate salts (MDP-Ca) with hydroxyapatite, providing a chemical link that may resist hydrolysis and reinforce interfacial durability [2,6,7,8]. This interaction, combined with mild self-etching demineralization, allows stable adhesion even in mineralized substrates such as sclerotic dentin.

From a materials-science perspective, this chemical interaction should be interpreted together with substrate-dependent factors such as surface energy, wettability, and the limited permeability of the hypermineralized dentin surface, all of which may influence interfacial adaptation and long-term bond durability [4,5,6,7,8].

Nevertheless, the superficial mineralized layer remains a physical barrier to infiltration and polymerization of adhesives. Mechanical pretreatments, such as air-abrasion with aluminum oxide particles, have been proposed to remove this surface layer, increase surface roughness, and improve wettability and energy, thus promoting monomer diffusion and interlocking [9,10]. Alternatively, bioactive air-abrasion using bioglass particles has been developed as a conservative surface treatment capable of releasing calcium and phosphate ions, theoretically promoting remineralization and enhancing substrate reactivity [11,12,13,14,15]. However, the actual effect of such bioactive pretreatments on adhesive performance remains uncertain, as residual particles or surface deposits may interfere with resin infiltration and reduce bond quality [11,13,14,15]. The composition of adhesive systems also plays a key role. Universal adhesives containing functional monomers such as MDP produce a mild demineralization while establishing chemical bonds with hydroxyapatite, resulting in improved stability [6,7,8,16,17,18]. In contrast, universal adhesives, while versatile, tend to be more hydrophilic and contain higher solvent content, making them more prone to water sorption, increased permeability, and hydrolytic degradation [16,17,18,19,20,21]. Consequently, the combined effect of adhesive chemistry and surface pretreatment is critical to achieving durable adhesion to sclerotic dentin.

In this context, surface pretreatment should not be regarded merely as a cleaning step, but as a strategy to modify the adhesive substrate and facilitate monomer diffusion into a low-permeability dentin surface [9,10,11,13,14,15].

Another important factor is the morphological heterogeneity within the same sclerotic area. Central zones, where tubules are fully occluded, differ significantly from peripheral areas that retain partial tubule patency [4,22]. This variation can influence resin infiltration and result in local differences in microtensile bond strength (µTBS), potentially affecting the reproducibility of results in laboratory studies. Clinically, the ability to achieve durable bonding to sclerotic dentin is essential for restoring non-carious cervical lesions, which are highly prevalent in adult populations [23,24]. Adequate adhesion in these cases ensures restoration retention, reduces marginal microleakage, and improves the longevity of resin-based restorations under functional and environmental stresses. Despite its clinical relevance, studies evaluating bonding strategies on naturally occurring sclerotic dentin remain limited because of the difficulty in obtaining extracted teeth fulfilling strict inclusion criteria and exhibiting homogeneous degrees of physiological sclerosis.

The aim of the present study was to assess the influence of surface conditioning, adhesive type, storage time, and dentin position (central versus peripheral) on the microtensile bond strength (µTBS) to naturally sclerotic dentin. The analysis focused on comparing the effects of mechanical (aluminum oxide) and bioactive (bioglass) air-abrasion on the bonding effectiveness and aging stability of different adhesive strategies. The null hypothesis was that neither the adhesive system, the type of surface pretreatment, the storage time, nor the position of the dentin substrate would have a statistically significant influence on the microtensile bond strength of sclerotic dentin.

## 2. Materials and Methods

### 2.1. Ethical Approval and Sample Selection

This in vitro study was conducted in accordance with the ethical principles of the Declaration of Helsinki and approved by the Research Ethics Committee of the Universitat de València (Ref. 2019-ODON-1031452; approval date: 9 May 2019). A total of sixteen human molars extracted primarily for periodontal reasons and, in a limited number of cases, for orthodontic indications were selected. Teeth were included if they fulfilled the following clinical criteria: they had been extracted from patients older than 45 years, had remained functional in the oral cavity until extraction, and presented no carious lesions, non-carious cervical lesions, restorations, or other structural defects affecting the crown. These inclusion criteria were established to increase the likelihood of obtaining teeth exhibiting physiological age-related dentin sclerosis [25,26]. The presence of naturally sclerotic dentin was not established before specimen preparation. Instead, after crown sectioning, the exposed dentin surface was examined macroscopically. Specimens exhibiting the characteristic translucent, shiny, and hypermineralized appearance traditionally associated with physiological dentin sclerosis were included in the study, whereas specimens not fulfilling these characteristics were excluded.

No validated grading scale or quantitative classification system was employed to determine the degree of dentin sclerosis. Consequently, specimen identification relied exclusively on the macroscopic characteristics of the exposed dentin surface after sectioning, following criteria commonly described for physiological age-related dentin sclerosis.

Sclerotic dentin specimens were selected according to strict macroscopic clinical criteria, including the presence of a characteristic glassy appearance, clinically evident hypermineralization, and a translucent surface appearance consistent with physiological dentin sclerosis. The extracted molars were obtained from patients older than 45 years in order to increase the probability of obtaining naturally occurring age-related sclerotic dentin in previous studies [4]. No validated grading scale or classification system was employed to quantify the severity of dentin sclerosis. Instead, specimen selection was based on clinically recognizable features that have been widely associated with naturally occurring sclerotic dentin in previous studies. All teeth exhibited sclerotic dentin identified by its characteristic glassy and hypermineralized appearance. After extraction, the teeth were cleaned of debris and stored in 0.1% thymol solution at 4 °C for no longer than three months before use. The crowns were sectioned perpendicular to the long axis using a low-speed diamond saw (Isomet 1000, Buehler Ltd., Lake Bluff, IL, USA) under continuous water irrigation to expose the sclerotic dentin surface. Following sectioning, the presence of a translucent and glassy dentin surface was considered consistent with physiological dentin sclerosis and served as the final criterion for specimen inclusion in the experimental procedures. The presence of a translucent and shiny surface confirmed sclerosis. The dentin was not polished or etched before the experimental procedures in order to preserve its natural morphology.

### 2.2. Experimental Design

Specimens were randomly assigned to nine experimental groups, combining three adhesives and three surface treatments (Table 1):

- Clearfil SE Bond 2 (Kuraray Noritake Dental, Tokyo, Japan): Group 1 (C): control.
- G-Premio Bond (GC Corporation, Tokyo, Japan): Group 2 (GP).
- iBond Universal (Kulzer GmbH, Hanau, Germany): Group 3 (I).
- Group 4 (C + SB): Al_2_O_3_ air-abrasion.
- Group 5 (GP + SB): Al_2_O_3_ air-abrasion.
- Group 6 (I + SB): Al_2_O_3_ air-abrasion.
- Group 7 (C + Sy): ProSylc^®^ air-abrasion.
- Group 8 (GP + Sy): ProSylc^®^ air-abrasion.
- Group 9 (I + Sy): ProSylc^®^ air-abrasion.

Due to the limited availability of extracted molars exhibiting naturally occurring physiological sclerosis and fulfilling all inclusion criteria, the number of teeth allocated to each experimental group was not completely homogeneous. All eligible specimens obtained during the collection period were included in order to maximize the available sample size for analysis.

### 2.3. Surface Pretreatment Protocols

Air-abrasion was performed for 5 s at 2 bars pressure from a distance of 10 mm, using an Aquacare (Velopex International, London, UK). For the aluminum oxide groups, 29 µm Al_2_O_3_ particles (ProCut^®^, OSSpray Ltd., London, UK) were used, whereas the bioactive groups were treated with 45 µm calcium sodium phosphosilicate glass (ProSylc^®^, Velopex International, London, UK). After air-abrasion, all surfaces were rinsed with distilled water for 15 s and gently air-dried to remove residual particles. No acid etching or mechanical polishing was performed prior to adhesive application.

For reproducibility, the air-abrasion protocol was standardized for time, pressure, and distance because these parameters may markedly affect surface morphology and subsequent adhesive spreading [9,10,27]. The selected air-abrasion parameters were intended to provide effective surface cleaning and modification while minimizing excessive substrate removal or structural damage to the sclerotic dentin surface. Although no quantitative surface characterization was performed, standardization of the air-abrasion conditions was considered essential to ensure comparability among the different pretreatment strategies evaluated.

### 2.4. Adhesive Application and Resin Build-Up

**Figure 1:**
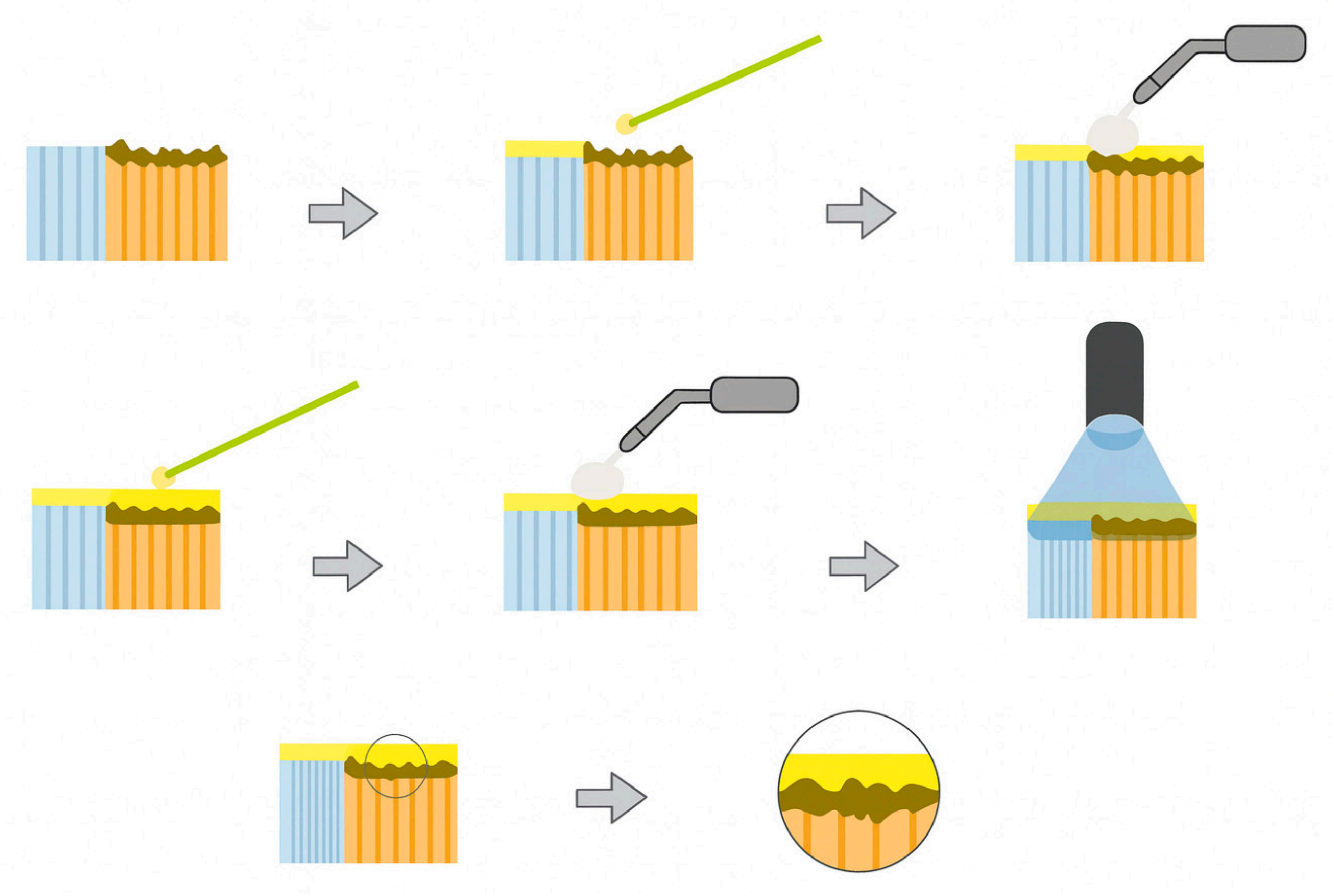
Diagram of the adhesive system application.

In Groups 1, 4, and 7, a two-step self-etch adhesive system (Clearfil SE Bond 2^®^, Kuraray Noritake Dental, Tokyo, Japan) was employed. The self-etch primer (Clearfil Self-Etch Primer^®^, Kuraray Noritake Dental, Tokyo, Japan) was applied using a small synthetic microbrush and actively rubbed onto the dentin surface for 20 s. Excess primer was removed by gentle air-drying with a dental syringe for 15 s at a distance of approximately 20 mm, ensuring a stable primer film on the treated surface. A saliva ejector was used throughout to prevent splatter. Subsequently, a layer of bonding resin (Clearfil Bond^®^) was applied with a microbrush and air-thinned until a uniform thin layer was obtained. Polymerization was then performed with a high-intensity LED curing unit for 20 s at 800 mW/cm^2^ (Bluephase 20i^®^, Ivoclar Vivadent, Schaan, Liechtenstein). For Groups 2, 5, and 8, which used a universal adhesive containing 10-MDP (G-Premio Bond^®^, GC Corp., Tokyo, Japan), and Groups 3, 6, and 9, which used a universal adhesive without 10-MDP (iBond U^®^, Kulzer GmbH, Hanau, Germany), the same self-etch application strategy was followed. Two consecutive coats of the universal adhesive were applied with a microbrush and actively rubbed for 20 s each, leaving the first coat uncured. The excess adhesive was then carefully removed using a saliva ejector to minimize splatter and air-dried vigorously for 15 s at a 20 mm distance. Finally, light curing was performed for 20 s at 800 mW/cm^2^ with a high-intensity LED curing unit (Bluephase 20i^®^, Ivoclar Vivadent, Schaan, Liechtenstein) (Figure 1).

Importantly, the universal adhesives were applied in two consecutive active coats, with the first coat left uncured, in order to improve surface wetting, solvent displacement, and monomer interaction with the sclerotic dentin substrate [16,17,18,19,20,21].

A light-cured composite resin (XBW G-aenial^®^, GC Corp., Tokyo, Japan) was applied directly from the syringe using a condenser instrument (Condenssa^®^, LM-Instruments Oy, Parainen, Finland), a spatula (Modella^®^, LM-Instruments Oy), and a fine brush (GC Corp., Tokyo, Japan) to obtain uniform specimens without air bubbles and with a diameter matching the dentin section of each molar. Each 2-mm increment was light-cured for 20 s at an irradiance of 1200 mW/cm^2^ using a high-intensity LED curing unit (Bluephase 20i^®^, Ivoclar Vivadent, Schaan, Liechtenstein). This layering procedure is commonly described in the literature as the build-up technique. The final composite block reached a total height of 6 mm, obtained by freehand layering of three consecutive increments. After polymerization of the last layer, a thin coat of glycerin gel (Liquid Strip^®^, Ivoclar Vivadent, Schaan, Liechtenstein) was applied to eliminate the oxygen-inhibited surface layer, followed by an additional 20-s light-curing cycle at 1200 mW/cm^2^. The selected material was a highly opaque, white-shade microhybrid composite, facilitating visual differentiation from the dental substrate. Its inorganic filler particles exhibited microhybrid size distribution and intrinsic fluorescence, which was particularly useful during the selection of microtensile beams, as natural enamel displays lower fluorescence intensity than the composite resin.

### 2.5. Beam Preparation and Classification

**Figure 2:**
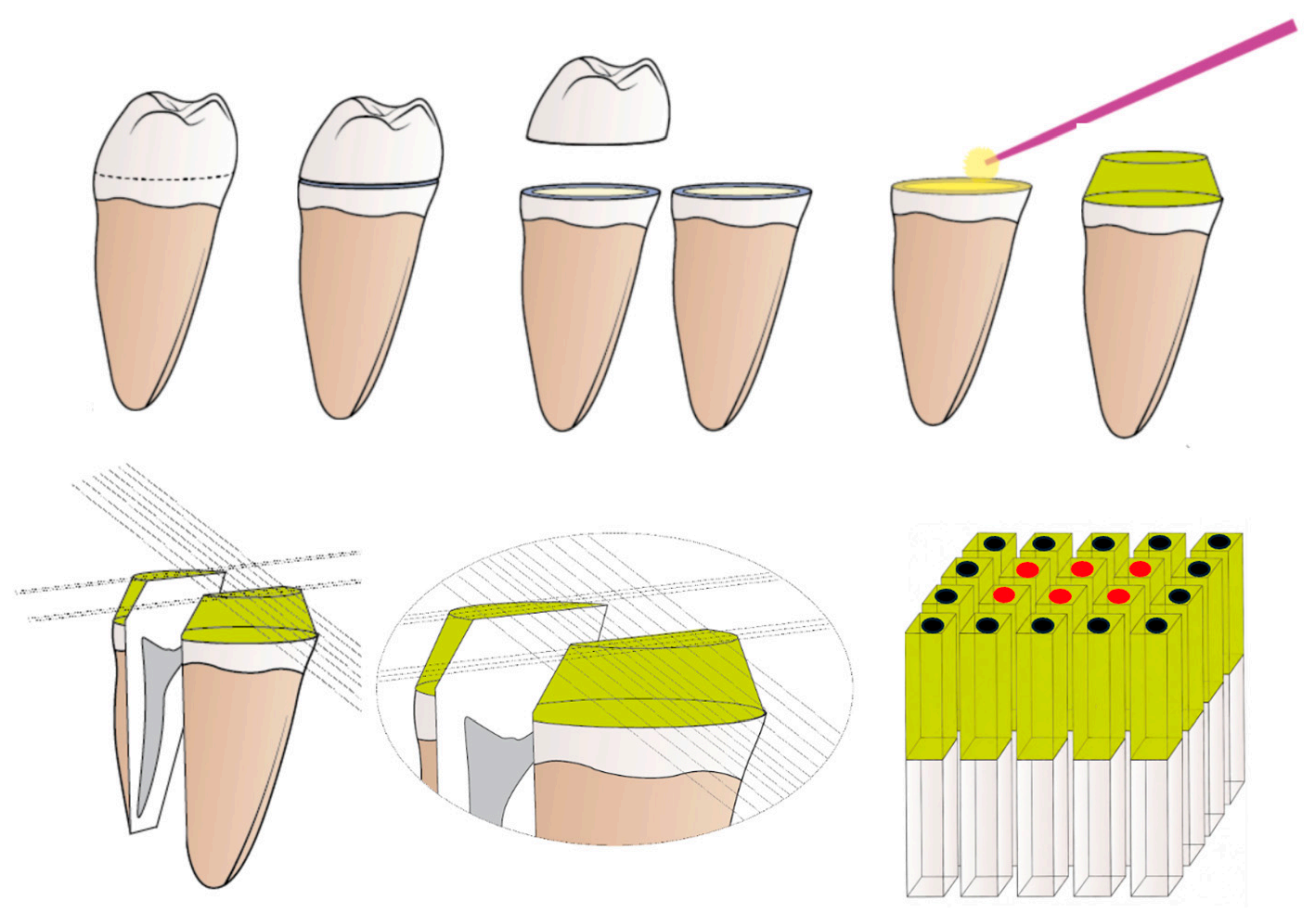
Schematic representation of tooth preparation and sectioning procedure for obtaining microtensile beams. The adhesive system application procedure. Peripheral beams marked with black dots, and central beams with red dots.

Following composite build-up, each restored tooth was sectioned longitudinally in both the mesiodistal and buccolingual directions using a low-speed diamond saw (Isomet 1000, Buehler Ltd., Lake Bluff, IL, USA) under continuous water cooling to obtain beams with an approximate cross-sectional area of 1 mm^2^. A total of 461 beams were obtained from the sixteen teeth. Before separating the individual beams from the remaining specimen, each beam was classified exclusively according to its position within the sectioning grid generated during the cutting procedure. Beams located in the central area of the grid were designated as central beams, whereas those located along the external margins of the grid were designated as peripheral beams (Figure 2). To preserve specimen identification throughout the experimental procedures, peripheral beams were marked with black ink and central beams with red ink before their separation from the specimen base. This classification was based solely on the spatial location of each beam within the sectioned specimen and was not intended to represent a direct assessment of the degree of dentin sclerosis, tubular occlusion, or mineralization. No microscopic examination, calibration procedure, or validated classification system was employed for this purpose. The beams were then randomly allocated to the corresponding storage and testing conditions according to the experimental design.

Each tooth was sectioned perpendicular to the bonded interface using the IsoMet^®^ 1000 precision cutter (Buehler Ltd., Lake Bluff, IL, USA) saw under water cooling to obtain beams of approximately 0.95–1.00 mm^2^ cross-sectional area. A total of 461 beams were obtained from the sixteen teeth (approximately 28–30 per tooth). Beams were classified according to their position within the sclerotic area: 1. Central beams: corresponding to the central area, the most mineralized and occluded region of sclerosis. 2. Peripheral beams: those located in the outermost region, at the perimeter of the sample grid (Figure 2).

### 2.6. Aging Protocol

Beams from each group were divided into two subgroups: 1. Immediate testing (24 h): after 24 h of water storage at 37 °C. 2. Aged testing (6–9 months): after long-term storage in distilled water at 37 °C, with weekly renewal. This design enabled assessment of both short-term and hydrolytic stability of the adhesive interfaces (Table 2). Long-term water storage at 37 °C was selected because it remains one of the most widely accepted laboratory aging methods for evaluating the hydrolytic degradation of resin–dentin bonds under in vitro conditions. Water aging may affect both the adhesive resin matrix and the collagen network within the hybrid layer, thereby providing information regarding the durability of the adhesive interface over time. The weekly renewal of the storage medium was performed to minimize saturation phenomena and maintain a stable aging environment throughout the experimental period. The selected aging protocol was intended to simulate long-term hydrolytic challenges and to provide a more clinically relevant estimation of bond durability than immediate bond strength measurements alone.

### 2.7. Microtensile Bond Strength Testing

Each beam was glued to a Bencor^®^ Multi-T Testing device (Danville Materials, San Ramon, CA, USA) using Zapit^®^ glue (Dental Ventures of America, Corona, CA, USA) and subjected to tensile load in a universal testing machine (LMT 100^®^, LAM Technologies, Florence, Italy) at a crosshead speed of 0.5 mm/min until failure. The bond strength (MPa) was calculated by dividing the load at failure (N) by the bonded area (mm^2^) measured with a digital caliper (Mitutoyo^®^, Tokyo, Japan). The values for each beam were recorded and grouped according to adhesive, surface treatment, storage time, and beam position.

Each tested beam was considered an individual bonded interface for mechanical evaluation purposes, allowing the influence of the different experimental factors on bond strength to be assessed at the specimen level. The microtensile test configuration was selected because of its ability to generate a relatively homogeneous stress distribution at the adhesive interface and its high sensitivity for detecting differences among adhesive strategies and dentin substrates.

### 2.8. Failure Mode and Statistical Analysis

Fractured specimens were observed under a stereomicroscope (Leica M125^®^, Leica Microsystems, Wetzlar, Germany) at ×40 magnification to determine the failure mode as adhesive, mixed, or cohesive in dentin or composite. Representative fractured specimens were qualitatively examined by optical microscopy to provide a general description of the fractured interfaces. No quantitative morphological analysis or standardized evaluation of the hybrid layer was performed. Representative specimens from each group were additionally examined using optical microscopy under magnification 6x to assess the hybrid layer and dentinal tubule characteristics morphology.

Data normality and homogeneity of variance were confirmed with Shapiro–Wilk and Levene tests, respectively. A four-way multifactorial ANOVA was performed (factors: adhesive system × surface treatment × storage time × beam position), followed by Tukey’s post-hoc test (α = 0.05). Statistical power was confirmed using G*Power 3.1 (Heinrich Heine Universität, Düsseldorf, Germany), and all analyses were conducted with IBM SPSS Statistics v27.0 (IBM Corp., Armonk, NY, USA). This statistical model was selected to account for the multifactorial nature of the experiment and to avoid attributing substrate- or aging-dependent effects exclusively to the adhesive material. Microtensile bond strength values were analyzed considering each tested beam as an individual analytical observation. Since several beams were obtained from each tooth, complete statistical independence among specimens originating from the same substrate cannot be assumed. Therefore, the potential clustering effect associated with the hierarchical structure of the data should be considered when interpreting the results.

## 3. Results

The multifactorial ANOVA confirmed significant main effects for adhesive system and surface treatment (*p* < 0.001), as well as an interaction between adhesive type and treatment (*p* < 0.05). Neither storage time nor beam position independently affected µTBS (*p* > 0.05), but relevant intra-group differences were identified when both variables were analyzed simultaneously.

At 24 h, Clearfil SE Bond 2 (C) achieved the highest control bond strength (34.8 ± 5.2 MPa). The application of Al_2_O_3_ air-abrasion (C + SB) produced a significant increase to 53.4 ± 6.1 MPa (*p* < 0.001, Tukey test), representing a 53% enhancement compared with the untreated control. The ProSylc^®^ treatment (C + Sy) resulted in slightly lower values (31.9 ± 4.9 MPa, *p* > 0.05), confirming that bioglass abrasion did not improve micromechanical retention. For G-Premio Bond, the control (GP) exhibited lower initial µTBS (24.6 ± 4.2 MPa). Al_2_O_3_ air-abrasion (GP + SB) significantly increased values to 29.1 ± 4.7 MPa (*p* = 0.021), while ProSylc^®^ (GP + Sy) did not differ from the control (25.8 ± 4.1 MPa, *p* > 0.05). iBond Universal showed the weakest bonding performance overall (range 20–24 MPa), and neither Al_2_O_3_ nor ProSylc^®^ conditioning significantly affected µTBS (*p* > 0.05).

**Figure 3:**
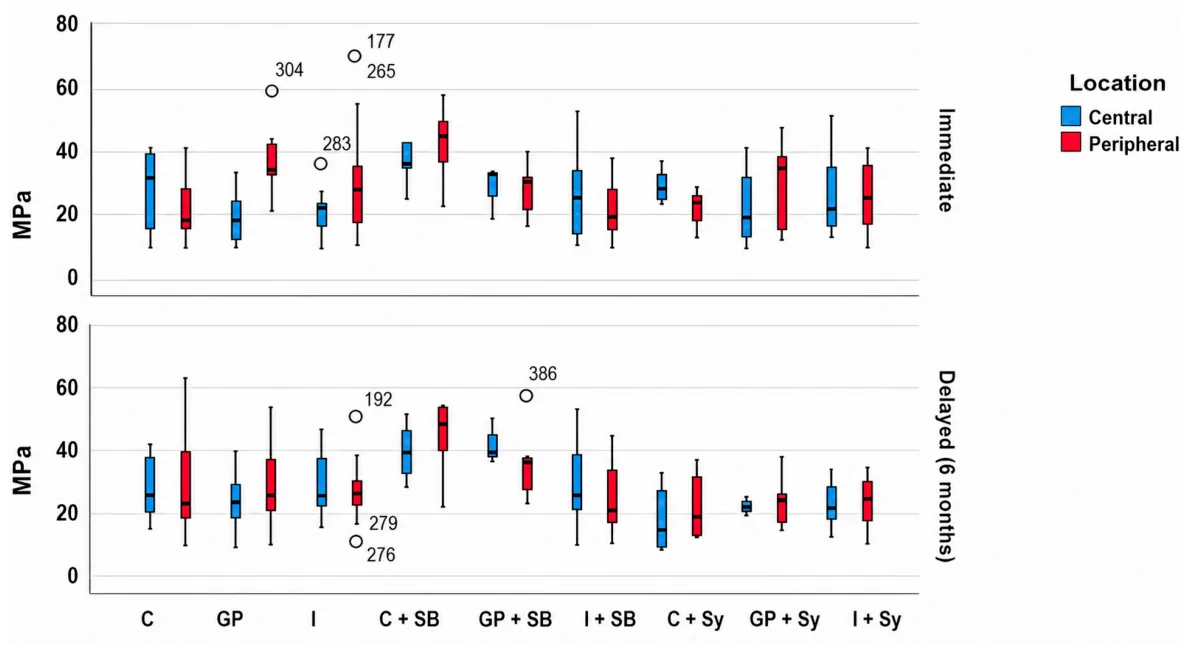
Distribution of F/S values according to the adhesive system (C: Clearfil; GP: G-Premio Bond; I: iBond; C + SB: Clearfil + 29 µm Al_2_O_3_ air-abrasion; GP + SB: G-Premio Bond + Al_2_O_3_ air-abrasion; I + SB: iBond + Al_2_O_3_ air-abrasion; C + Sy: Clearfil + ProSylc^®^ air-abrasion; GP + Sy: G-Premio Bond + ProSylc^®^ air-abrasion; I + Sy: iBond + ProSylc^®^ air-abrasion), considering beam location (central or peripheral) and testing time (upper panel: immediate; lower panel: delayed, 6 months). In the figure, the circles indicate the outliers.

After 6 months of water storage, most groups maintained their bond strengths, confirming good hydrolytic stability of the interfaces. Only the Clearfil SE Bond 2 control group exhibited a small but statistically significant increase in µTBS (Δ = +1.4 MPa, *p* = 0.033, paired t-test), indicating possible post-curing polymerization or gradual stress relaxation within the adhesive layer. Universal systems showed slight but non-significant decreases after aging, especially in ProSylc^®^-treated specimens (GP + Sy: from 25.8 ± 4.1 MPa to 23.5 ± 3.9 MPa, *p* = 0.081), possibly due to increased water sorption and plasticization of their more hydrophilic matrices (Figure 3).

While the global analysis showed no positional effect (*p* = 0.312), notable intra-group differences were observed. In G-Premio Bond, µTBS values were significantly higher for peripheral beams (33.2 ± 4.1 MPa) than for central beams (21.7 ± 3.9 MPa) (*p* = 0.002). This difference was not detected in Clearfil SE Bond 2 or iBond Universal (*p* > 0.05). When combining position and treatment, Al_2_O_3_ air-abrasion consistently increased µTBS across all adhesives and beam positions, with a proportionally greater effect in central beams, especially for Clearfil SE Bond 2 (+60% over control). ProSylc^®^ produced irregular results, likely due to incomplete removal of mineralized deposits or residual particle embedding (Table 3). The positional behavior of G-Premio Bond suggests that the heterogeneity of sclerotic dentin may influence adhesive performance even within the same lesion, particularly for more technique-sensitive universal adhesives.

**Figure 4:**
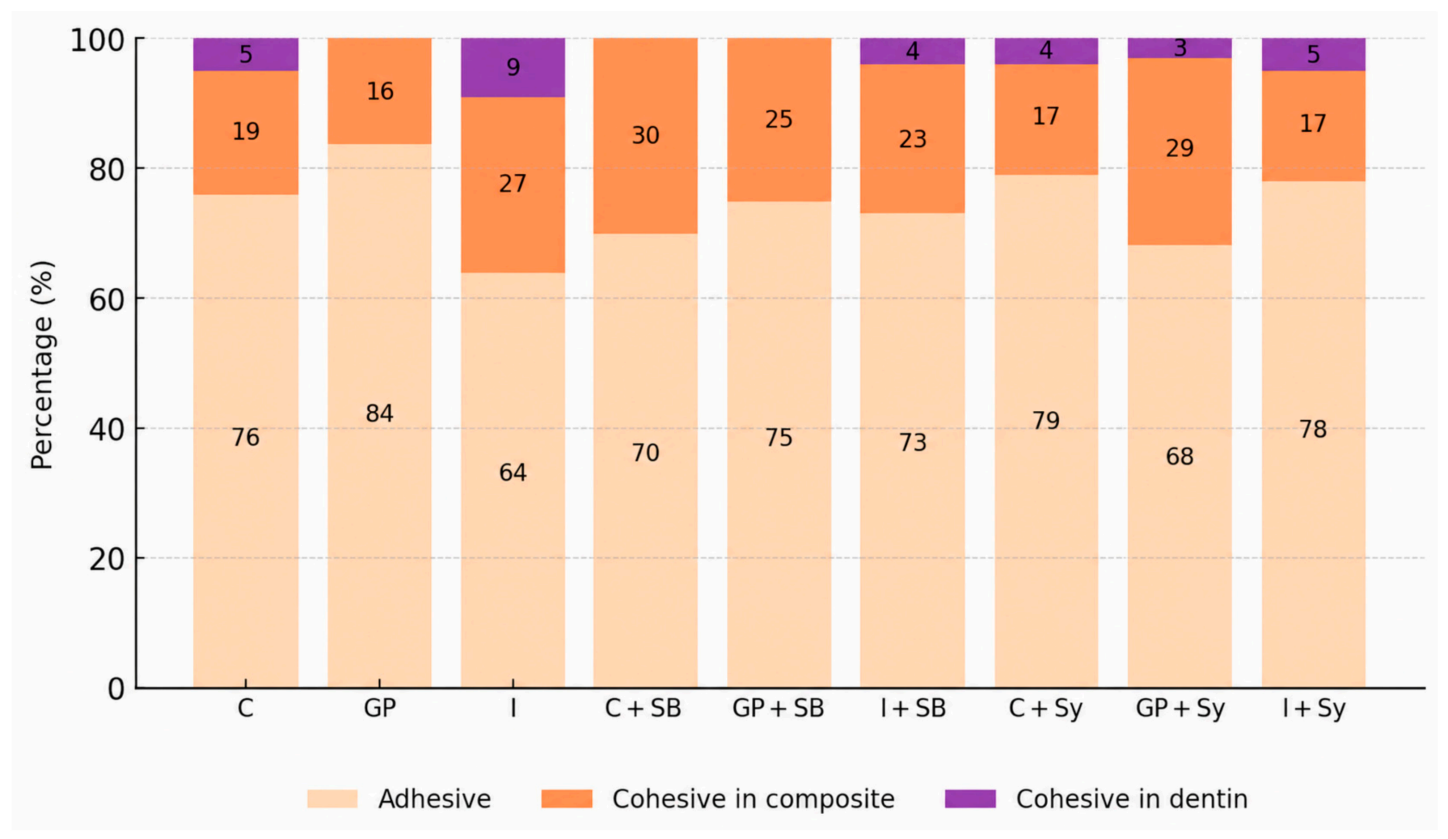
Percentage of each failure type according to the adhesive system used (C: Clearfil; GP: G-Premio Bond; I: iBond; C + SB: Clearfil + Al_2_O_3_ air-abrasion; GP + SB: G-Premio Bond + Al_2_O_3_ air-abrasion; I + SB: iBond + Al_2_O_3_ air-abrasion; C + Sy: Clearfil + ProSylc^®^ air-abrasion; GP + Sy: G-Premio Bond + ProSylc^®^ air-abrasion; I + Sy: iBond + ProSylc^®^ air-abrasion).

Failure mode analysis showed predominantly adhesive failures (73%), followed by mixed (22%) whereas cohesive failures within dentin or composite resin represented less than 5% of the tested specimens and cohesive (<5%) failures. Al_2_O_3_-treated groups showed a higher proportion of mixed failures, which may be indicative of improved interfacial integrity and enhanced micromechanical interaction between the adhesive and the conditioned dentin substrate (Figure 4). Al_2_O_3_-treated groups showed more mixed failures, consistent with improved interface integrity. SEM revealed open tubules and uniform hybrid layers in air-abraded groups, whereas ProSylc^®^-treated surfaces exhibited particle residues and irregular hybridization Optical microscopy observations suggested more homogeneous interfacial patterns in the aluminum oxide air-abrasion groups, whereas ProSylc^®^-treated specimens exhibited irregular interfacial morphology and residual surface deposits that could potentially interfere with adhesive infiltration Qualitative observations performed under optical microscopy revealed differences in the general appearance of the fractured interfaces among the experimental groups. However, because no representative optical micrographs or complementary high-resolution morphological analyses were performed, these observations should be interpreted only as descriptive findings and not as definitive evidence of differences in interfacial morphology or hybrid layer quality.

No pre-testing failures occurred, and coefficients of variation remained below 20%, confirming reproducibility. Overall, Clearfil SE Bond 2 achieved the highest and most stable bond strengths, particularly after Al_2_O_3_ air-abrasion. G-Premio Bond benefited moderately but was sensitive to substrate position, and iBond Universal remained the least effective regardless of treatment or aging.

## 4. Discussion

This study evaluated the effect of different surface treatments, adhesive systems, storage times, and substrate position on the microtensile bond strength (µTBS) to sclerotic dentin. The results demonstrated that both the type of adhesive and the surface treatment significantly influenced µTBS (*p* < 0.001), while neither storage time nor beam position exerted independent main effects. However, relevant intra-group differences emerged when these two variables were analyzed simultaneously. These findings allow a partial rejection of the null hypothesis (H_0_), since the adhesive type and surface treatment significantly affected bond strength, whereas storage time and substrate position only exerted secondary or interaction-dependent influences.

Consistent with previous studies, bonding effectiveness depends largely on the adhesive’s ability to infiltrate the sclerotic dentin and form a continuous and stable hybrid layer [1,2,3,4,5]. The self-etch adhesive containing a functional MDP monomer achieved the highest bond strengths, particularly after Al_2_O_3_ air-abrasion, which increased µTBS by more than 50% compared with the untreated control. This finding supports prior evidence showing that mechanical removal or modification of the hypermineralized surface layer may enhance wettability and monomer diffusion, thereby improving micromechanical interlocking [9,10,27]. In contrast, bioglass air-abrasion failed to produce significant improvements in any group, in agreement with studies reporting that bioactive glass particles may promote mineral deposition but can also interfere with resin infiltration when residual particles remain on the dentin surface [11,12,13,14,15]. Although bioglass exhibits potential remineralizing properties, its application before bonding on sclerotic dentin may not be advantageous under the present protocol, particularly if residual surface particles are not adequately removed. These findings are consistent with the conclusions of a recent systematic review, which suggested that mechanical surface pretreatments may improve bonding to naturally sclerotic dentin, although the available evidence remains limited and further high-quality studies are still required [28].

The differences among adhesives reflect the influence of their chemical composition and hydrophilicity. Universal adhesives, which contain higher proportions of solvents and are more hydrophilic, are prone to water sorption, increased permeability, and polymer plasticization [16,17,18,19,20,21,29,30,31,32]. This could explain the slight reduction in µTBS observed after storage in water, particularly in bioglass-treated groups, where surface moisture may have exacerbated hydrolytic degradation. The intermediate universal adhesive benefited moderately from Al_2_O_3_ air-abrasion but showed marked sensitivity to substrate position. µTBS values were significantly higher in peripheral beams than in central ones, in line with previous descriptions of increased mineral density and tubular occlusion in central areas of sclerotic dentin [4,22]. This microstructural difference likely limits resin infiltration and micromechanical retention at the lesion’s center. Conversely, the peripheral regions, where some tubules remain open, allow deeper hybridization and stronger bonding.

These results confirm that substrate microstructure is a decisive factor, even within the same specimen. Although qualitative optical microscopy suggested differences in the appearance of the fractured interfaces among the experimental groups, these observations should be interpreted with caution because no representative optical micrographs or complementary high-resolution morphological analyses were available. Consequently, the mechanisms proposed to explain the bond strength results should be regarded as possible interpretations rather than direct morphological evidence. The observed failure patterns and interfacial behavior suggest improved interaction after Al_2_O_3_ air-abrasion, whereas bioglass-treated surfaces may have been affected by residual particles and irregular hybridization. Such findings are consistent with studies emphasizing that adequate surface conditioning is essential to optimize micromechanical interaction between dentin and adhesive [9,10,11,13,14,15,27]. The hydrolytic stability observed after six months of storage supports the long-term durability of adhesives containing functional monomers capable of forming stable bonds with hydroxyapatite, such as MDP [2,6,7,8,29,30]. This behavior may result from the formation of MDP-Ca salts and nanolayered structures, which have been associated with improved resistance to chemical and mechanical degradation [6,7,8]. Moreover, the slight increase in µTBS detected in the self-etch control group could be related to post-curing polymerization or gradual stress relaxation within the adhesive layer, improving interfacial cohesion over time [31,32]. Long-term water storage remains one of the most frequently employed laboratory aging methods for evaluating the durability of resin–dentin bonds. The hydrolytic degradation occurring during water storage may affect both the adhesive resin matrix and the collagen network within the hybrid layer, thereby providing a more clinically relevant estimation of long-term adhesive performance than immediate bond strength measurements alone.

The stability observed after storage may be partly related to the lower permeability of the sclerotic substrate and to chemical interactions mediated by functional monomers; however, this mechanism should be interpreted cautiously because MDP-Ca nanolayering was not directly assessed in the present study [6,7,8,29,30,31,32]. These observations should be interpreted as indirect evidence of substrate-dependent adhesive behavior rather than as definitive microstructural characterization, since no quantitative roughness, contact-angle, nanoleakage, or high-resolution interfacial mapping was performed. From a clinical perspective, these findings suggest that Al_2_O_3_ particle air-abrasion is an effective pretreatment to enhance bonding to sclerotic dentin, especially when combined with self-etch strategies containing stable functional monomers [9,10,23,24]. Conversely, bioactive conditioning with bioglass, though conceptually appealing, appears unsuitable for this substrate under the tested protocol due to its negative effect on micromechanical retention and the potential presence of residual particles compromising adhesion [11,12,13,14,15]. The clinical implications of this study are significant for non-carious cervical lesions, where sclerotic dentin is the predominant substrate [23,24]. A protocol combining gentle mechanical cleaning with a mild self-etch adhesive containing functional monomers may optimize both initial bond strength and clinical longevity of composite restorations exposed to functional and environmental stresses. The lack of improvement after bioglass treatment does not rule out its bioactive potential, but suggests that, under the present protocol, residual particles or surface deposits may have interfered with resin infiltration and micromechanical interlocking [11,12,13,14,15]. The present findings are in agreement with recent clinical and laboratory evidence indicating that the characteristics of the sclerotic substrate and the selected surface conditioning protocol play a decisive role in the long-term success of restorations placed in non-carious cervical lesions [33,34]. Future studies should combine mechanical testing with SEM and EDS analyses to better characterize the effects of surface pretreatments on naturally sclerotic dentin. Nevertheless, because no sound dentin control group was included, the present findings should be interpreted as comparisons among different adhesive protocols applied to naturally sclerotic dentin rather than as direct comparisons between healthy and sclerotic substrates.

Clinically, these findings are relevant because sclerotic dentin in non-carious cervical lesions is not a uniform substrate: central hypermineralized areas and peripheral regions may respond differently to the same adhesive strategy.

Regarding the limitations, it must be acknowledged that this was an in vitro study, which does not fully reproduce the dynamic oral environment, including thermal fluctuations, occlusal loading, enzymatic activity, or long-term fatigue. An additional limitation of the present study is that physiological dentin sclerosis was confirmed only after specimen preparation by macroscopic evaluation of the exposed dentin surface. Although this approach follows the methodology adopted in previous laboratory investigations, it does not constitute a validated diagnostic method for identifying naturally sclerotic dentin before specimen preparation. Future studies incorporating complementary imaging techniques or validated diagnostic criteria would further strengthen specimen characterization. In addition, no quantitative roughness, contact-angle, nanoleakage, or high-resolution interfacial mapping was performed, so the proposed mechanisms should be interpreted as explanatory hypotheses rather than direct measurements [17,18,19,29,30,31,32]. Nevertheless, the controlled experimental design ensured reproducibility, as indicated by coefficients of variation below 20%, consistent with laboratory standards. Future research should include in vivo or in situ studies to validate these findings under clinical conditions and assess long-term performance. Another limitation of the present study is the absence of a sound dentin control group. The experimental design was intentionally focused on comparing different adhesive strategies exclusively on naturally occurring sclerotic dentin, since this substrate represents the clinical condition of interest. Consequently, the present results allow comparison among the evaluated adhesive protocols within sclerotic dentin, but they do not permit direct comparison with normal dentin or quantification of the specific effect of dentin sclerosis on bond strength. Future studies should include both healthy and naturally sclerotic dentin under identical experimental conditions to further clarify the influence of substrate characteristics on adhesive performance.

## 5. Conclusions

Within the limitations of this in vitro study, both the adhesive system and the surface treatment significantly affected the microtensile bond strength to sclerotic dentin. Air-abrasion with aluminum oxide proved to be the most effective conditioning method, particularly when combined with self-etch adhesives containing functional monomers such as MDP, yielding the highest and most stable bond strengths. In contrast, bioglass air-abrasion did not enhance bonding and produced irregular interfaces, while beam position only influenced performance in one universal adhesive, with higher values in peripheral areas. The combination of aluminum oxide air-abrasion and an MDP-containing self-etch adhesive demonstrated the most favorable bonding behavior under the experimental conditions evaluated in the present investigation. Nevertheless, these findings should be interpreted within the limitations inherent to the in vitro design and the statistical structure of the dataset. Further studies including larger sample sizes, complementary surface characterization techniques, and alternative statistical approaches are required to confirm the present findings and to establish their potential clinical implications. Overall, Al_2_O_3_ air-abrasion followed by a self-etch MDP-based adhesive appears to be a reliable and durable approach for achieving optimal adhesion to sclerotic dentin in non-carious cervical lesions.

## Figures and Tables

**Table 1:** Experimental groups and surface treatment protocols.

Group	Name	Procedure
1	C	No surface pretreatment + two-step self-etch adhesive (Clearfil™ SE Bond 2, Kuraray Noritake Dental, Tokyo, Japan).
2	GP	No surface pretreatment + universal adhesive containing 10-MDP (G-Premio Bond™, GC Corp., Tokyo, Japan).
3	I	No surface pretreatment + universal adhesive without 10-MDP (iBond^®^ Universal, Kulzer GmbH, Hanau, Germany).
4	C + SB	Surface pretreatment with aluminum oxide (Procutt^®^, OSSpray Ltd., UK) + two-step self-etch adhesive (Clearfil™ SE Bond 2).
5	GP + SB	Surface pretreatment with aluminum oxide (Procutt^®^) + universal adhesive containing 10-MDP (G-Premio Bond™).
6	I + SB	Surface pretreatment with aluminum oxide (Procutt^®^) + universal adhesive without 10-MDP (iBond^®^ Universal).
7	C + Sy	Surface pretreatment with Bioglass 45S5 (BAG) powder (Sylc^®^, Velopex International, London, UK) + two-step self-etch adhesive (Clearfil™ SE Bond 2).
8	GP + Sy	Surface pretreatment with Bioglass 45S5 (BAG) powder (Sylc^®^) + universal adhesive containing 10-MDP (G-Premio Bond™).
9	I + Sy	Surface pretreatment with Bioglass 45S5 (BAG) powder (Sylc^®^) + universal adhesive without 10-MDP (iBond^®^ Universal).

**Table 2:** Distribution of specimen molars and microtensile beams per experimental group.

Group	Specimen Molar	Beams	Peripheral	Central	Immediate	Aged
G1	1 and 2	59	45	14	26	27
G2	3, 4, and 5	77	50	27	50	27
G3	6, 7, and 8	92	61	31	63	29
G4	9	23	14	9	12	11
G5	10	20	14	6	10	10
G6	11 and 12	57	37	20	30	27
G7	13	31	15	16	16	15
G8	14	34	25	9	16	18
G9	15 and 16	60	38	22	30	30

The unequal number of specimen molars among groups reflects the limited availability of extracted teeth exhibiting natural physiological sclerosis and fulfilling all inclusion criteria.

**Table 3:** Descriptive statistics of the variable F/S (MPa) according to the adhesive system (GROUP).

Group	N	Mean (MPa)	SD	95% CI Lower	95% CI Upper	Minimum	Maximum
C	75	26.2 ^b^	12.2	23.2	28.8	9.33	63.64
GP	50	27.4 ^b^	11.6	25.0	31.6	9.46	57.42
I	74	26.8 ^b^	11.7	24.4	29.8	9.20	69.15
C + SB	23	40.2 ^a^	10.3	36.3	45.1	22.15	57.16
GP + SB	20	32.9 ^a^^b^	9.8	27.6	36.7	16.21	54.27
I + SB	52	25.3 ^b^	11.1	21.4	27.6	9.52	52.28
C + Sy	24	22.5 ^b^	8.5	18.7	25.8	9.26	36.39
GP + Sy	31	24.4 ^b^	10.3	21.9	29.4	9.20	46.67
I + Sy	58	24.5 ^b^	9.3	22.0	26.9	9.54	50.00

(C: Clearfil SE Bond 2; GP: G-Premio Bond; I: iBond Universal; C + SB: Clearfil SE Bond 2 + Al_2_O_3_ air-abrasion; GP + SB: G-Premio Bond + Al_2_O_3_ air-abrasion; I + SB: iBond Universal + Al_2_O_3_ air-abrasion; C + Sy: Clearfil SE Bond 2 + ProSylc^®^ bioglass abrasion; GP + Sy: G-Premio Bond + ProSylc^®^ bioglass abrasion; I + Sy: iBond Universal + ProSylc^®^ bioglass abrasion). Superscript letters (a, b) indicate statistically significant differences between mean values (Tukey test, *p* < 0.05).

## Data Availability

The data supporting the findings of this study are available from the corresponding author upon reasonable request.
